# A Pilot Study Examining Speed of Processing Training (SPT) to Improve Processing Speed in Persons With Multiple Sclerosis

**DOI:** 10.3389/fneur.2018.00685

**Published:** 2018-08-27

**Authors:** Nancy D. Chiaravalloti, Yael Goverover, Silvana L. Costa, John DeLuca

**Affiliations:** ^1^Neuropsychology and Neuroscience Laboratory, Kessler Foundation, West Orange, NJ, United States; ^2^Department of Physical Medicine and Rehabilitation, Medical School, Rutgers, The State University of New Jersey, Newark, NJ, United States; ^3^Department of Occupational Therapy, New York University, New York, NY, United States; ^4^Department of Neurology, Medical School, Rutgers, The State University of New Jersey, Newark, NJ, United States

**Keywords:** cognitive rehabilitation, cognitive functions, daily life activities, multiple sclerosis, disease course

## Abstract

**Background:** Individuals with Multiple Sclerosis (MS) have significant impairments in processing speed (PS) and such impairments may underlie other cognitive deficits common in MS and limit performance of everyday life activities.

**Objective:** To examine the efficacy of a computerized PS intervention, Speed of Processing Training (SPT), in persons with MS on PS, memory and everyday activities.

**Methods:** Twenty-one individuals with clinically definite MS and an objectively assessed impairment in PS were included in a controlled randomized clinical trial, randomly assigned to a treatment group or a control group. Participants were assessed prior to and within 1 week of completing the treatment. Outcome measures included traditional neuropsychological tests measuring PS and memory, and an assessment of PS in daily life activities.

**Results:** The treatment group showed a significant improvement on neuropsychological tests of PS and new learning and memory. A significant improvement was additionally noted in the treatment group on measures of PS in everyday life. These changes were not observed in the control group.

**Conclusions:** Results provide preliminary data in support of SPT in treating PS deficits in persons with MS. Additional research is needed with larger samples and more comprehensive outcome measures.

## Introduction

Multiple Sclerosis (MS) is the most common nontraumatic neurological illness in young and middle-aged adults (1). Sensorial, motor, cognitive, and psychiatric problems are common, with high variability in symptom presentation (1). Processing speed (PS) is widely recognized as the single greatest cognitive deficit in MS (2, 3).

PS affects all cognitive domains and tends to be correlated with verbal abilities (4), working memory (WM) (5) and new learning and memory (NLM) (6, 7). Thus, a deficit in PS may be the source of deficits in other areas of cognition. As an example, PS has been shown to correlate with new learning abilities in persons with MS (8). The term new learning refers to the process by which new, to-be-learned information is initial acquired (9); this is in contrast to the term memory, referring to the retrieval of previously learned information at a later point in time (6).

Cognitive deficits in MS also negatively impact employment (10, 11), social activities (12), driving (13, 14), and the ability to complete everyday tasks (15, 16). Recent findings also indicate PS to play an important role in benefit from cognitive rehabilitation (CR) targeting other areas of cognition. For instance, Chiaravalloti and DeLuca (17) showed that persons with intact PS benefitted from memory treatment, while those with impaired PS did not (17). Thus an effective means of treating PS deficits in MS must be identified.

Existing CR programs in MS have been focused on attention, WM, communication skills and NLM [see (18, 19)]. However, only one study to our knowledge specifically addresses PS deficits in MS. Hancock et al. (20) provided preliminary evidence for an at-home CR program for PS deficits in persons with MS who self-reported cognitive impairment. The active training group improved on a PS/WM measure following treatment relative to controls. However, the study's high drop-out rate and inclusion of persons who self-reported PS cognitive deficits may present external and internal validity issues.

CR protocols designed to train multiple cognitive skills, including PS, have also been developed and tested (21–24), with improved cognitive performance noted following treatment. For example, improvement has been shown in PS and other targeted cognitive domains following RehaCom, which persist over time [e.g., (25)]. However, since such rehabilitations programs were designed to train multiple cognitive skills, the relative focus on PS and the impact of treatment on PS specifically is unknown.

Speed of Processing Training (SPT) is a computerized treatment designed to improve PS, with substantial supporting evidence in normal aging on laboratory measures of PS and everyday functioning [e.g., (26)]. Ball et al. (26) examined data from six studies applying SPT to aging, demonstrating consistent improvements on lab-based tests of PS and everyday functioning, maintained at 2-year follow-up (27). Longitudinal analyses showed treatment effects to remain 5 and 10-years later (27). Given its efficacy in aging, we conducted an initial pilot study examining the efficacy of SPT in MS, hypothesizing that MS participants treated with SPT would show improved performance on neuropsychological tests of PS and NLM, as well as an objective test of daily functioning compared to a control group.

## Methods

### Participants

Twenty-one participants [Treatment (TX) *n* = 12, Control (CTL) *n* = 9] with clinically definite MS (28) were recruited from MS Clinics, advertisements and through the Kessler Foundation database of research participants. There were no significant differences between groups on demographic (age, education, gender, pre-morbid IQ) or disease related variables (disease duration, subtype; Table 1). Groups were also similar on pre-treatment PS and verbal abilities.

**Table 1:** Demographic and disease characteristics by group (treatment vs. control).

	**Control (*n* = 9) M(SD)**	**Treatment (*n* = 12) M(SD)**	t Statistic (unless indicated)^*^
Age (years)	52.11 (7.3)	46.42 (7.4)	*t*_(19)_ = −1.75, *p* = 0.1
Education (years)	15.33 (2.6)	15.25 (2.6)	*t*_(19)_ = −0.07, *p* = 0.94
Percent female	67%	75%	X^2^ = 0.176, *p =* 0.68
Percent right handed	88%	92%	X^2^ = 0.09, *p =* 0.76
Months since diagnosis	41 (26.9)	152 (59.2)	*t*_(19)_ = 2.40, *p* = 0.1
Disease subtype relapsing remitting	100%	100%	n/a
WASI vocabulary (pre-morbid IQ estimate)	49.56 (12.0)	48.42 (12.9)	*t*_(19)_ = 0.84, *p* = 0.84
SDMT Z-score at baseline (baseline PS ability)	−2.38 (1.26)	−1.91 (.79)	*t*_(19)_ = 0.30, *p* = 0.3
Token test	31.22 (2.3)	31.25 (2.6)	*t*_(19)_ = 0.98, *p* = 0.98
**DISEASE MODIFYING THERAPIES**			
None	3	3	
Copaxone	4	4	
Avonex	1	0	
Bestaseron	1	1	
Aubagio	0	1	
Rebif	0	1	
Tysabri	0	1	
Unknown	–	1	

* *All comparisons non-significant*.

### Inclusion criteria

Participants were 18–65 years of age and free of exacerbations and steroid use for at least 1 month. All participants demonstrated baseline *impaired PS*, defined as performance 1.5 standard deviations or more below the mean of published normative data on the oral Symbol Digit Modalities Test (SDMT) (29). Participants were excluded if they had a major psychiatric disorder, substance abuse, evidence of significant vision impairment from diplopia, nystagmus or scotomas upon testing (corrected vision in worse eye >20/60 assessed with Snellen Eye Test) or impaired language comprehension on the Token Test.

### Design

**Figure 1:**
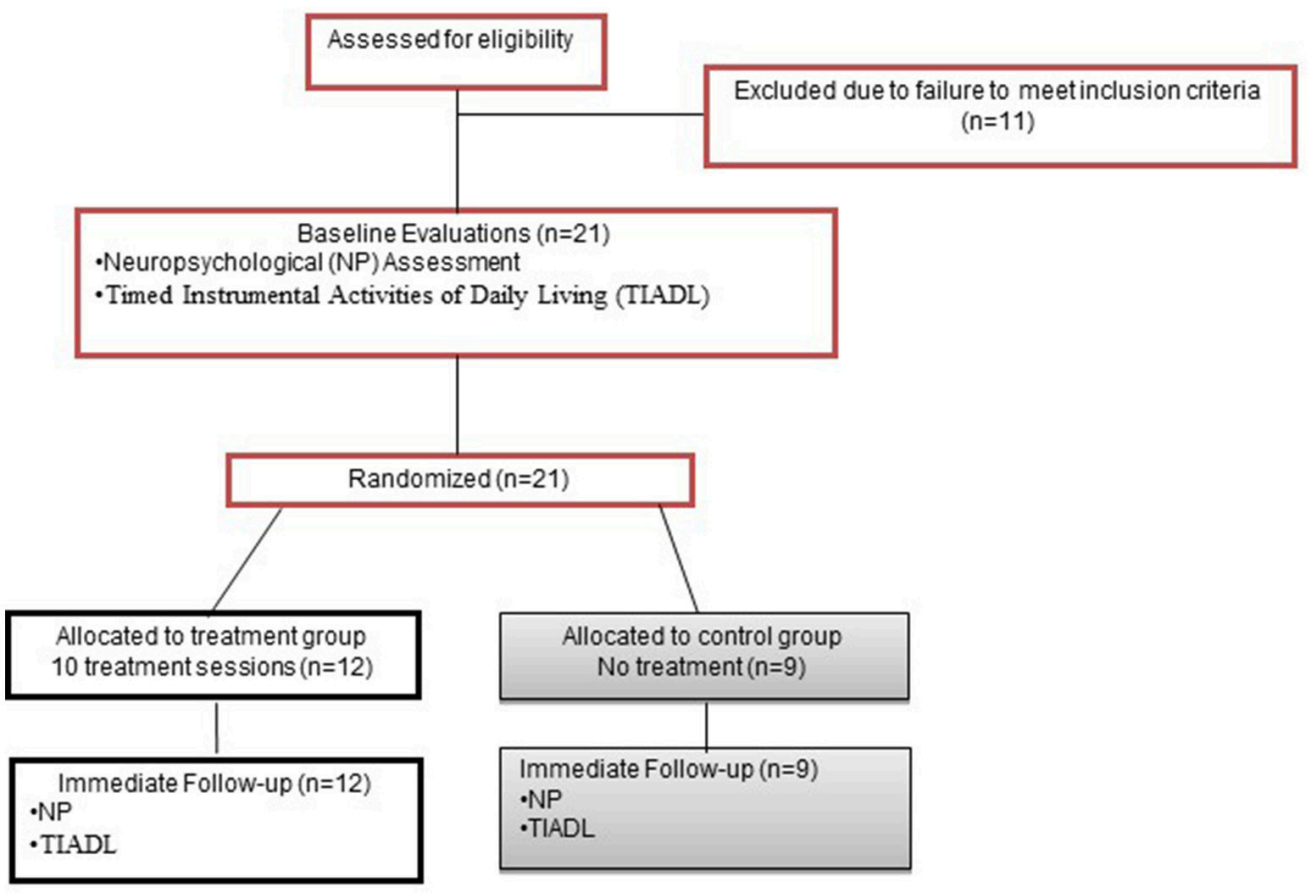
Experimental overview.

This RCT used a 5-week, parallel groups design (Figure 1). Potential participants underwent a 2-part screening consisting of initial telephone screen for age, injury type and date, neurological history, and current medications; and in-person screening for psychiatric and substance abuse history, visual acuity, language comprehension and PS abilities.

The groups were assigned via 1:1 randomization using a computerized random number generator. Treatment allocation was concealed. The individual responsible for group assignment was not otherwise involved in data collection, and group assignment was verified by a second individual via duplicate copy of the randomization table generated prior to the initiation of data collection. Only the person administering treatment knew group assignment. All other study personnel were blinded, assured through several mechanisms (Therapists and evaluators were always different and were not able to communicate directly about participants). Participants were informed that they had a 50% chance of being assigned to treatment.

Once qualifying, participants underwent baseline evaluation, including neuropsychological (NP) assessment and the Timed Instrumental Activities of Daily Living Test [TIADL (30)], performed in one session. Tests were administered in a standard order for all participants. Within 1 week of completing treatment, participants completed the same evaluation with alternate forms. The same evaluator conducted baseline and immediate follow-up evaluations wherever possible.

### Treatment protocol

The ***Treatment Condition*** consisted of 10 computerized training sessions over a 5-week period similar to the work of Ball et al. (31). Initial sessions involve practice on three types of tasks presented on a computer (simple speed of processing, divided attention, and selective attention), with three different central demands (detection, identification, same/different). Training is customized to each participant's ability; an individual's entry point into SPT is determined by current level of PS, evaluated as the speed of stimulus presentation at which the person can correctly identify the stimulus 75% of the time. If this threshold is 30 ms or greater, SPT begins at the most basic level. Training sessions lasted approximately 30–45 min each depending on self-reported fatigue or an observable drop in performance.

#### In task I

Level I participants practice a single discrimination task at progressively faster speeds. The task is composed of either target present or absent, target identification, or same/different judgments. Training continues with increasingly more complex discrimination tasks until the participant can perform the target identification task correctly 75% of the time, at exposure duration of 17 ms, at which time the participant progresses to Level II.

#### Task II

Level II requires the participant to perform one of the discrimination tasks described above, and simultaneously locate a peripheral target. The demand of the center target can be varied, as described above, and the peripheral task demand is changed by decreasing or increasing the distance of the peripheral target from the center target (32). The process of progressing from near peripheral targets to far targets is repeated, at faster speeds, and with increasing difficulty of the center task. This is repeated until the participant can perform both the foveal identification task and the peripheral localization task (at the furthest eccentricity) with 75% accuracy, at a speed of 50 ms or less. Once this level is achieved, the participant progresses to Level III.

#### Task III

Level III (selective attention training) requires the participant to perform a discrimination task and locate a peripheral target embedded among distracters. It begins with a display duration near threshold, with the peripheral target placed at a near eccentricity. When the participant is able to perform the selected task correctly 75% of the time, a more demanding task is introduced by manipulating the complexity of the discrimination task, the display duration and/or target eccentricity. Practice continues until 75% correct performance is achieved at a 120 ms exposure, with peripheral targets at the most extreme eccentricity.

The ***Control Condition*** was a no treatment control condition.

### Outcome measures

Neuropsychological assessments were scheduled according to the participants' availability, the majority of which were scheduled for either 10 a.m. or 1 p.m. Every effort was made to schedule a given participant's assessments (baseline and follow-up) at the same time of day.

The *primary outcome* was a measure of PS, *Digit Symbol Coding Subtest* from the Wechsler Adult Intelligence Scale-III (WAIS-III) (33). Subjects are presented with a key of nine digit-symbol pairings at the top of a page and given 120 s to write the corresponding symbol for 133 digits. The raw score is the total number of correct pairings. The subtest has high test-retest reliability (*r* = 0.84) and good construct validity.

*Secondary outcomes* were (1) additional measures of PS, *Letter Comparison* (LC) and *Pattern Comparison* (PC)(34) (2) the California Learning Verbal Test II (CVLT-II) (35), measuring the impact of changes in PS on NLM and (3) the TIADL, an objective test of speeded everyday life tasks.

*LC* is a measure of perceptual speed requiring the examinee to determine whether two strings of printed letters (3, 6, or 9 characters) are identical or different on two 30-s trials of 21 items. *PC* reflects the same task with non-verbal stimuli, two printed geometric line patterns. Examinees respond orally in LC and PC, with “s” for same or “d” for different.

The CVLT-II examines verbal NLM. A list of 16 words from four semantic categories is presented orally over five trials for free recall. Two alternate forms minimize carryover between testing sessions. The dependent variables (DV) were the learning slope across the 5 learning trials (LS) and Short Delay Free Recall (SDFR)(35).

The TIADL is a performance-based measure of functional activities uses real everyday items comprising five tasks sampling common instrumental activities of daily living: (1) communication: finding a number in a phone book, (2) finance: counting change using coins, (3) nutrition: locating and reading ingredients from a food can, (4) shopping: locating items on a shelf, and (5) medicine: locating and reading directions from medicine bottles. Scoring was determined by both accuracy and time to completion. To minimize the effects of motor deficits, stimulus materials are placed in the participant's hand immediately before the task begins (when applicable), and tasks require very simple motor ability. Each task has a maximum time limit of 2 min, with the exception of the communication task, which has a limit of 3 min. If a participant does not complete a task within the time limit, the task is terminated and maximum time is assigned. The TIADL has excellent test-retest reliability (*r* = 0.85) and criterion validity (19). A positive relationship has also been noted between TIADL performance and PS (30, 36).

### Statistical analyses

Due to the pilot nature of the current study an intend-to-treat design was not utilized. Analysis examining treatment effects utilized an ANCOVA with baseline performance as the covariate in each analysis. All analyses were performed with SPSS version 18 software. No interim data analyses were performed.

### Ethics and registration

All procedures were approved by an institutional review board and all participants provided written informed consent. The RCT is registered with clinicaltrials.gov (NCT01838824).

## Results

Recruitment ran from 1/31/2012-2/2/13. The trial ended with completion of external funding. 21 participants with clinically definite MS were randomly assigned to TX (*n* = 12) or CTL (*n* = 9), with no dropout.

### Treatment efficacy

#### Changes on neuropsychological tasks of PS

**Figure 2:**
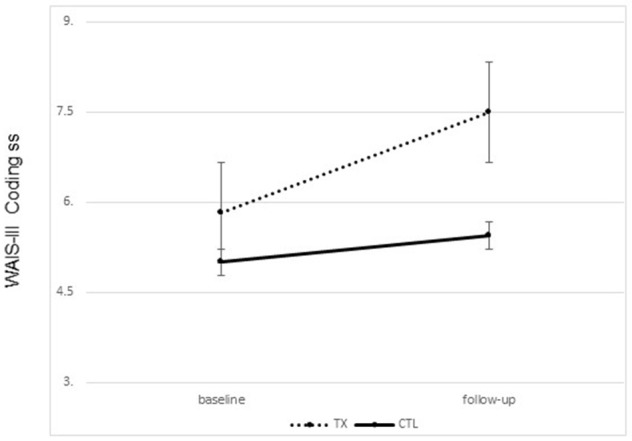
Changes on the WAIS-III coding subset scaled scores pre to post treatment by group (treatment vs. control; group means plus standard error of the mean).

Significant improvement was noted from pre- to post-treatment on the primary outcome, the WAIS-III Coding Subtest, in the treatment group only. Treated participants completed the WAIS-III Coding tasks faster following treatment than prior to treatment. No change was noted in the control group [*F*_(1, 21)_ = 2.72, *p* = 0.05, one tailed; partial Eta^2^ = 0.133, large effect; Figure 2]. Interestingly, 25% of our treated sample moved from an impaired score on the Coding subtest to a not impaired score following treatment, while 0% of the participants in the control group showed this change.

Albeit non-significant, a medium-large effect size was noted from pre- to post treatment on PC [*F*_(1, 21)_ = 2.16, *p* = 0.08, one tailed; partial Eta^2^ = 0.107, medium-large effect]; such that the treatment group showed more correct responses after treatment while the control group showed no change. No significant difference was noted from pre- to post-treatment on LC (partial Eta^2^ = 0.025; small effect) (Table 2).

**Table 2:** Performance on the neuropsychological tests.

	**Pre treatment**		**Post treatment**	
	**Mean (SD)**		**Mean (SD)**	
	**Control (*n* = 9)**	**Treatment (*n* = 12)**	**Control (*n* = 9)**	**Treatment (*n* = 12)**
Coding	5 (2.35)	5.83 (2.62)	5.44 (2.35)	7.50 (2.84)
Letter comparison	6.89 (1.88)	8.40 (4.09)	6.78 (2.37)	8.13 (2.65)
Pattern comparison	12.78 (3.18)	13.46 (3.34)	12.06 (4.28)	13.71 (3.18)
CVLT slope	1.12 (0.43)	1.13 (0.66)	0.99 (0.40)	1.17 (0.61)
CVLT sDFR	8.67 (4.36)	8.08 (3.50)	6.56 (3.54)	8.75 (4.27)

*SD, standard deviation; CVLT, California Learning Verbal Test II; SDFR, short delay free recall*.

#### Changes in learning and memory abilities

**Figure 3:**
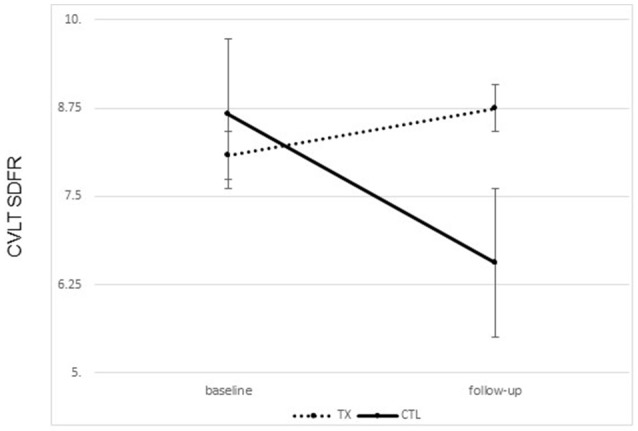
Changes on the California Learning Verbal Test II short delay Free Recall (CVLT SDFR) from ore to post treatment by group (treatment vs. control group means plus standard error of the mean).

A significant improvement was noted from pre- to post-treatment on the CVLT-II SDFR in the treatment group only. That is, participants who completed treatment were able to recall more words following treatment than prior to treatment [*F*_(1, 21)_ = 4.93, *p* = 0.015, one tailed; partial Eta^2^ = 0.215, large effect; Figure 3]. No change was noted in the control group.

There were no significant differences between the groups on the CVLT learning slope from pre- to post-treatment (partial Eta^2^ = 0.029; small effect) (Table 2).

#### Changes in everyday life functioning

**Figure 4:**
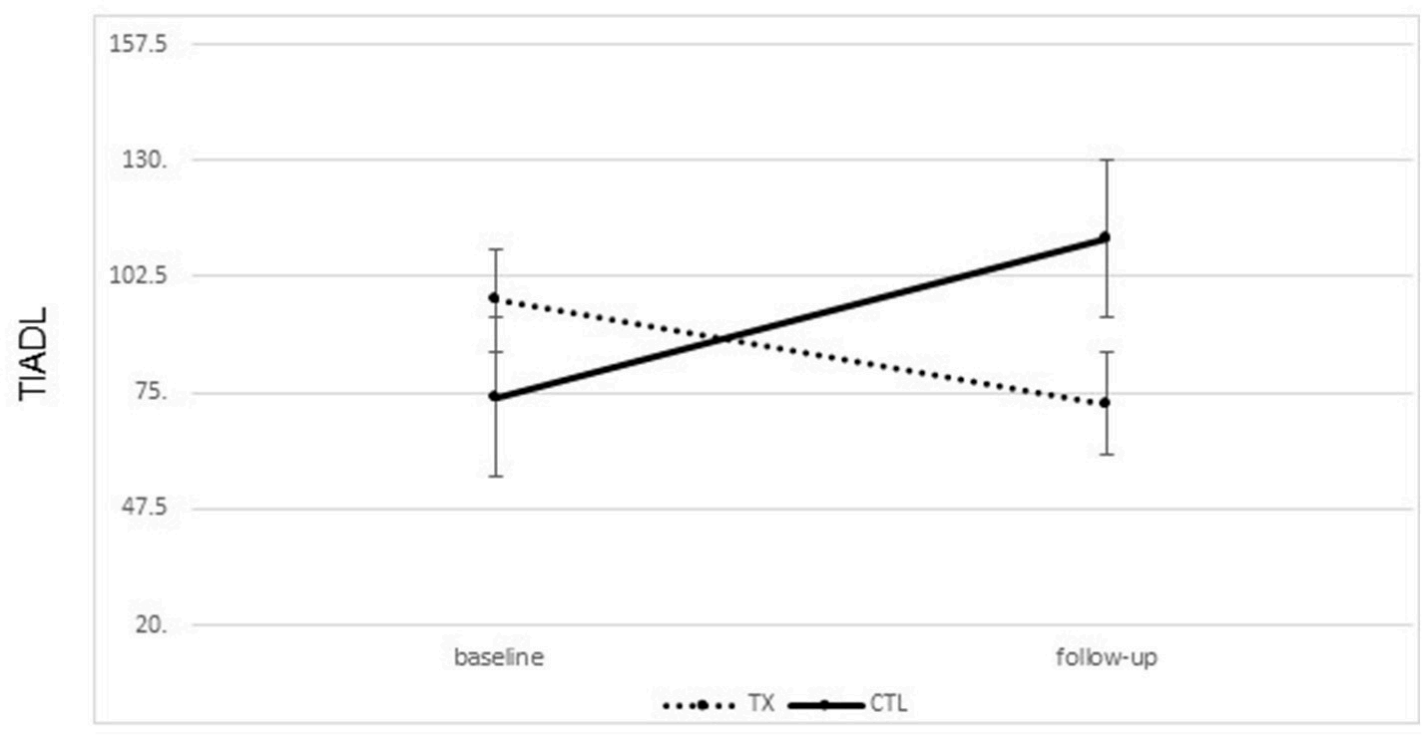
Changes on the timed instrumental activities of daily living phonebook task from pre to post treatment (mean seconds to complete task; lower score is better; bars represent standard error of the mean).

A significant improvement was noted from pre- to post-treatment on the TIADL with the treatment group showing improved accuracy and speed on the TIADL following treatment compared to the control group [*F*_(1, 19)_ = 8.4. *p* = 0.01, Eta^2^ = 0.33], noted specifically on the finding a number in a phonebook task [*F*_(1, 19)_ = 6.3. *p* = 0.02, Eta^2^ = 0.27] (Figure 4). In addition, treatment participants were able to locate and read ingredients on a can of food significantly faster and more accurately following treatment than prior to treatment compared to the control group [*F*_(1, 19)_ = 5.55, *p* = 0.03; partial Eta^2^ = 0.246; large effect; Table 3].

**Table 3:** Performance on the timed instrumental activities of daily living (scores incorporates speed and accuracy) by group.

	**Pre treatment**			**Post treatment**				
	**Mean (SD)**			**Mean (SD)**			**ANCOVA interaction**	
	**Treatment group (*n* = 12)**	**Control group (*n* = 9)**	***t***	**Treatment group (*n* = 12)**	**Control group (*n* = 9)**	***F*_(1, 19)_**	***p* ≤**	**Partial Eta^2^**
TIADL z-score	0.11 (1.02)	−0.02 (0.53)	0.15	0.38 (0.76)	−0.23 (0.45)	**8.4**	**0.01**	**0.33**
Communication-finding phone numb Z-SCORE	−0.25 (0.94)	0.20 (1.06)	1.06	0.47 (1.07)	−0.31 (0.84)	**6.3**	**0.02**	**0.27**
Finances-counting change	0.23 (1.1)	−0.17 (0.87)	−0.94	0.09 (1.1)	−0.07 (0.96)	0.21	ns	0.01
Nutrition-locate and read ingredients on cans of food	0.35 (1.9)	−0.17 (0.87)	−0.94	0.62 (1.0)	−0.41 (0.73)	**5.5**	**0.03**	**0.24**
Shopping-locate food items on a shelf	−0.13 (1.3)	0.09 (0.65)	0.51	0.13 (0.68)	−0.10 (1.2)	0.43	ns	0.02
Medicine-locate and read Instructions	0.07 (1.1)	−0.05 (0.96)	−0.26	0.35 (1.4)	−0.26 (0.28)	2.5	ns	0.12

*Bold values are significant results*.

There were no significant group differences on any other TIADL metric.

## Discussion

Results indicated that relative to the control group, the group receiving SPT showed significant improvements on neuropsychological tests of PS, NLM and daily life. These findings support our hypotheses and emphasize the importance of assessing the impact of a CR protocol not only within the domain targeted for treatment, but also on higher order cognitive functions reliant on basic cognition and on cognitive functioning in daily life. While CR studies continue to accumulate in MS, few studies examine the impact of treatment on daily life (19). This is despite the fact that multiple experts have stressed the importance of evaluating post-CR changes on daily life in addition to the gold standard of neuropsychological testing [e.g., (37)].

A significant treatment effect was noted from before to after treatment on the primary PS outcome, the Coding subtest of the WAIS-III. Albeit non-significant, a large effect size was also observed on a secondary measure of PS ability, PC. There are several possible explanations for these positive findings. Costa et al. (3) proposed the tri-factor model of PS in MS, hypothesizing that PS difficulties in MS are associated with three independent deficits: a sensorial speed deficit (sensorial processing), a cognitive speed deficit (information manipulation), and a motor speed deficit (oral or written motor response, or performance of eye movements for a visual search). Related to this theory, performance on Coding and PC is dependent on how quickly and accurately an individual is able to execute these three information processing stages: (1) process visual information; (2) manipulate the information and plan a response; (3) provide a response (written on Coding and oral on PC) and subsequently move on to the next target stimulus (perform saccadic eye movements). It is well known that these three components of information processing are often impaired in MS (3). The SPT trains these three stages individually. In SPT Level I, individuals are trained to process visual information at faster presentation rates (visual speed training); in SPT Levels II and III individuals are trained to manipulate information within shorter periods of time (cognitive speed training) and perform faster saccadic eye movements toward the periphery (motor speed training). Thus, one can hypothesize that SPT is effective for the rehabilitation of PS deficits in MS because it trains sensorial, cognitive and motor speed, all of which are fundamental to the performance of visual PS tests and often impaired in MS [see (3)]. More research is needed to explore the impact of SPT on visual, cognitive and motor speed individually and fully understand its impact on performance of visual PS tasks.

Importantly, the current results add to the growing body of literature supporting the efficacy of cognitive training in persons with MS (19). Given the frequency with which slowed PS is noted in the MS population, it is vital that we identify effective means for addressing this deficit specifically (3). Shatil et al. (38), compared a group completing training with CogniFit to a no treatment control group, noting the cognitive training to be associated with increased naming speed and speed of information recall. Vogt et al. (39) noted that 45 MS participants completing training with BrainStim saw improved fatigue, working memory abilities and PS post-treatment. Perez-Martin et al. (40) similarly showed an improvement in memory, fluency and PS following treatment with a computer assisted neuropsychological training program in 30 treated participants as compared with 32 participants assigned to a control condition. The RehaCom cognitive training software has similarly demonstrated efficacy in its ability to treat PS specifically in several samples of persons with MS [e.g., (41–44)]. Similarly, Covey et al. (45) showed a n-back training program to results in improved functioning on working memory, PS, complex attention and reasoning. Pedulla et al. (46) utilized an at-home training program, the COGNI-TRAcK in 28 persons with MS, noting significant improvement on verbal learning and recall, verbal fluency, sustained attention, concentration and PS. Substantial gains are thus noted following cognitive rehabilitation for PS specifically.

A second major finding of the current study is the significant improvement of treated participants on a NLM test, the CVLT-II, with a significant treatment effect noted on SDFR. It is important to note that the same pattern of results was noted on the CVLT-II slope, albeit non-significant, with the treatment group showing an improvement post treatment and the control group showing a decline. It is notable that a CR protocol directly addressing PS resulted in a change in NLM. This highlights the role of PS as a more basic level of processing that clearly impacts NLM (3). In fact, Chiaravalloti and DeLuca (17) demonstrated a memory rehabilitation treatment to be effective in persons with MS with intact PS abilities, but not persons with MS with impaired PS abilities, hypothesizing two distinct groups of MS memory impaired participants–one with a hippocampally-mediated memory deficit and the other with a memory deficit resulting from impaired PS. The current pattern of results lends support to this hypothesis, highlighting the intricate connection between PS and learning and memory in MS.

Finally, results also indicated a significant impact of treatment on performance of the TIADL total score and subtests of the phonebook task and locating and reading ingredients on a can of food. Locating and reading instructions on a medicine bottle did not reach significance, but showed a medium effect size. These three tasks are similar because all three require participants to quickly scan the visual field in order to locate the required information. The noted improvement may thus have been impacted by improved visual scanning post-treatment ***in addition to*** improved cognitive PS, consistent with the tri-factor model of PS in MS (3).

Despite the positive impact of SPT on PS, NLM and speed-based tasks of daily life in this study, there are some limitations. First, generalization is limited by the small sample. Future work would benefit from the inclusion of larger samples to further document the impact of treatment on daily life. Second, this study is limited by the outcome measures utilized. To truly understand the impact of SPT on PS in MS, one must include more outcome measures focused on PS without a motor component. Measures of multiple higher order cognitive functions (e.g., memory and executive functioning) would be a goal for future research. Third, future work should include an active control group to control for non-specific factors related to participation in treatment. Future work should additionally evaluate the impact of SPT on daily life from the perspective of both the patient and the significant other. While self-report measures have been criticized for being biased by depression, awareness and other symptomatology, they provide important information about the perception of the patient and family. Finally, the maintenance of the treatment effect over time is important to evaluate for any cognitive rehabilitation program and should be considered in future work.

The current findings support the application of SPT to treat PS deficits in MS. A particular strength of the current study is the use of a well-studied technique previously shown to improve PS in healthy aging with participants diagnosed with MS who have objectively verified PS deficits. This study is a first step in designating a CR program directed to treat PS in MS.

## Author contributions

NC acquired funding, directed study execution, conducted data analysis, led interpretation and drafted manuscript. YG assisted with data analysis, interpretation and manuscript preparation specifically in regard to everyday life data (TIADL). SC assisted with data collection, analysis, interpretation, and manuscript preparation. JD assisted with obtaining funding, analysing data, interpreting data, and manuscript preparation.

### Conflict of interest statement

The authors declare that the research was conducted in the absence of any commercial or financial relationships that could be construed as a potential conflict of interest.

## Abbreviations

CLT: Control
CR: Cognitive rehabilitation
CVLT II: California Learning Verbal Test-2nd Edition
DV: Dependent variable
LC: Letter comparison
LS: Learning trials
Ms: Milliseconds
MS: Multiple Sclerosis
NLM: New learning and memory
NP: Neuropsychological
PC: Pattern comparison
PS: Processing speed
SDFR: Short Delay Free Recall
SDMT: Symbol Digit Modalities Test
SPT: Speed of Processing Training
TIADL: Timed Instrumental Activities of Daily Living Test
TX: Treatment
WAIS–III: Wechsler Adult Intelligence Scale-III.
